# Arbuscular mycorrhizal fungi enhance soil carbon sequestration in the coalfields, northwest China

**DOI:** 10.1038/srep34336

**Published:** 2016-10-17

**Authors:** Zhi-Gang Wang, Yin-Li Bi, Bin Jiang, Yryszhan Zhakypbek, Su-Ping Peng, Wen-Wen Liu, Hao Liu

**Affiliations:** 1 State Key Laboratory of Coal Resources and Safe Mining, China University of Mining and Technology (Beijing), Beijing 100083, China; 2 Kazakh National Research Technical University named after K.I. Satpayev, Almaty, 050013, Kazakhstan

## Abstract

Carbon storage is affected by photosynthesis (P_n_) and soil respiration (R_s_), which have been studied extensively in natural and agricultural systems. However, the effects of P_n_ and R_s_ on carbon storages in the presence of arbuscular mycorrhizal fungi (AMF) in coalfields remain unclear. A field experiment was established in 2014 in Shendong coal mining subsidence area. The treatments comprised two inoculation levels (inoculated with or without 100 g AMF inoculums per seedlings) and four plant species [wild cherry (*Prunus discadenia Koebne* L.), cerasus humilis *(Prunus dictyneura Diels* L.), shiny leaf Yellow horn (*Xanthoceras sorbifolium Bunge* L.) and apricot (*Armeniaca sibirica* L.)]. AMF increased P_n_ of four species ranging from 15.3% to 33.1% and carbon storage, averaged by 17.2% compared to controls. Soil organic carbon (OC), easily extractable glomalin-relation soil protein (EE-GRSP), and total glomalin-relation soil protein (T-GRSP) were significantly increased by AMF treatment. The effect of AMF on the sensitivity of R_s_ depended on soil temperature. The results highlighted the exponential models to explain the responses of R_s_ to soil temperature, and for the first time quantified AMF caused carbon sequestration and R_s_. Thus, to our knowledge, AMF is beneficial to ecosystems through facilitating carbon conservation in coalfield soils.

Carbon storage depends on the balance between carbon sequestration by plant P_n_ and carbon release to atmosphere through R_s_^1^,^2^. Therefore, if plant P_n_ or R_s_ is altered, carbon balance will be affected. R_s_, especially seasonal variations, are significantly affected by soil temperature and moisure^3^,^4^. However, the impact of soil microbes on R_s_ or carbon storage remains unclear. So far, quantified contribution of soil microbes, especially AMF has not been reported yet. AMF are obligate plant symbionts that associates with the roots of more than 80% vascular land plants, which significantly enhance long-term success of mine site reclamation^5^. Studies showed that AMF are helpful for building up a productive, healthy, and sustainable post-mine land ecosystem with vegetation cover. The positive impact of AMF on reclaimed soil fertility and plant communities succession has been well documented^6^,^7^,^8^. However, few studies focused on the effects of AMF on R_s_ in post-mine land. A recent study conducted in Germany grassland communities reported that AMF stimulated R_s_ on pasture soil, leading to elevated CO_2_ level and temperature, with most carbon sequestered in belowground parts, making R_s_ an important component of carbon balance^9^,^10^. Factors such as soil moisture, soil temperature, CO_2_ enrichment, and precipitation changes were all affected R_s_. However, relevant studies remain scare and the complexity of various interactions that affect R_s_ (such as soil temperature vs moisture, soil temperature vs microbes, moisture vs microbes interactions) are still poorly understood.

R_s_ includes intergated CO_2_ flux of root respiration, mycorrhizal respiration (considered as part of autotrophic respiration), and heterotrophic respiration, which are controlled by carbon supply and temperature^9^,^10^. Carbon supply is an important R_s_ impact factor, which primarily depends on plant productivity and generally responds positively to CO_2_ enrichment with the increased P_n_^11^. Numerous studies confirmed that carbon storage depended on plant P_n_ and R_s_ via AMF in greenhouse or field experiments^7^,^9^,^10^,^11^.

AMF usually form symbiotic associations with trees in diverse forests. The fungi rely on host plant to obtain carbon supply and utilize 5–20% of the net photosynthate of the symbiotic system^11^. Global forest soil releases 24 Pg Carbon per year into atmosphere via CO_2_ efflux and generates CO_2_ from a widely variety of belowground organisms, with AMF as the dominant carbon source^12^. AMF as an important carbon and soil CO_2_ efflux source have been well studied under various temperature and moisture conditions^12^. A pulse-labeling experiment showed that in temperate prairie, about 4–6% photoassimilates were facilitated via AMF^10^. Simultaneously, AMF enhanced 16% soil CO_2_ efflux when *Lolium pernne* roots were colonized and 8% carbon supply was derived from AMF in a barley field^13^. To date, no studies have been carried out on the contribution of AMF on carbon storage in coal mining soil.

Respiratory quotient (*Q*_10_) is defined as the temperature sensitivity of R_s_ which is derived from substrate availability, which is the respiration variation ratio when soil temperature rises 10 °C. *Q*_10_ is largely affected by a series of environmental factors, such as soil physicochemical properties and soil moisture, etc. However, few studies have quantified the effects of AMF on apparent *Q*_10_ from different tree species. Thus, knowledge about the function of AMF on R_s_, P_n_, and carbon storage is insufficient^14^.

Notably, CO_2_ concentration indirectly influences R_s_ and carbon allocation from host species to fungus, suggesting that root participates in carbon fixation^15^,^16^,^17^. Most colonized plants enhanced productivity or nutritional status of the plant symbioses system when exposed to higher CO_2_ concentration^18^. In addition, mycorrhizal hyphae assisted carbon re-distribution from aboveground parts to roots via utilizing the photosynthate and provides multiple adherent agents such as extracellular polymer, and amino acid, etc than non-colonized plants^19^. Briefly, AMF enhance soil carbon proportion in higher CO_2_ environment^20^,^21^,^22^. However, lacking a better understanding of these responses in coal mining soil limited to prediction soil carbon storage when AMF existed in current scenarios.

Thus, the experiment planted wild cherry, cerasus humilis, shiny leaf Yellowh orn and apricot in coal mining soil and half seedlings were inoculated with AMF inoculums. Based on the experimental results, two hypotheses was proposed that: (i) plant species preferentially facilitate their photosynthetic ability and increase R_s_ rates, leading to different carbon storage amount; and (ii) soil temperature rather than soil moisture is sensitive to R_s_ due to the extreme drought. The future goal is to investigate the effect of AMF on carbon sequestration in coal mining fields.

## Results

### P_n_ and R_s_ temporal dynamics

**Figure 1:**
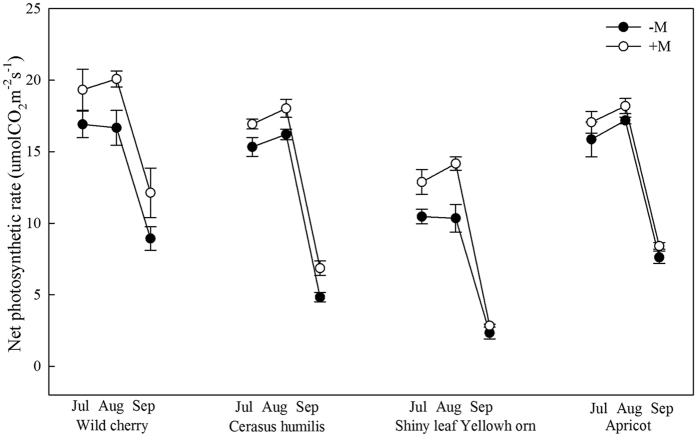
Net photosynthetic rate of four species in different growth stage. Average monthly variation in P_n_ (n = 18) in wild cherry, cerasus humilis, shiny leaf Yellowh orn, and apricot from July to September. Values represented means ± SE. −M and +M denoted without and with AMF inoculation.

P_n_ was significantly greater with AMF inoculation in all the four species from July to September (Fig. 1). Specifically, AMF treatment increased P_n_ in wild cherry, cerasus humilis, shiny leaf Yellowh orn, and apricot by 21.2%, 15.0%, 53.1%, and 7.4%, respectively. Meanwhile, P_n_ in all four tree species increased from July to August and then strikingly decreased from August to September (Fig. 1).

**Figure 2:**
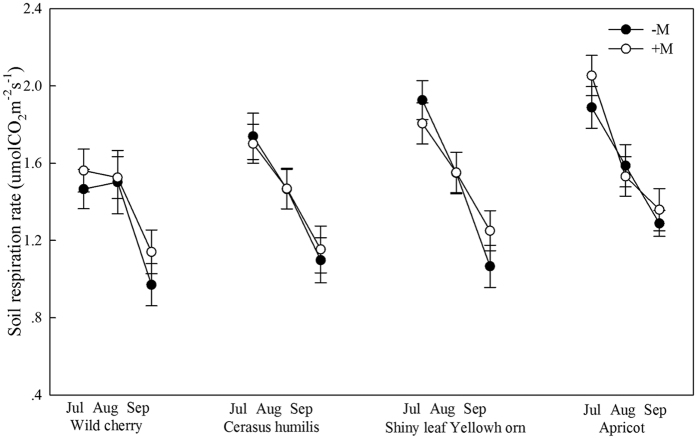
Soil respiration rate of four species in different growth stage. Average monthly variation in R_s_ (n = 18) in wild cherry, cerasus humilis, shiny leaf Yellowh orn, and apricot from July to September. Values represented means ± SE. −M and +M denoted without and with AMF inoculation.

In all tree species, R_s_ was significantly decreased from July to September, with slight decrease from July to August and reduced sudden reduction of 21.3–26.9% in September (Fig. 2).

### Cumulative carbon measures

**Figure 3:**
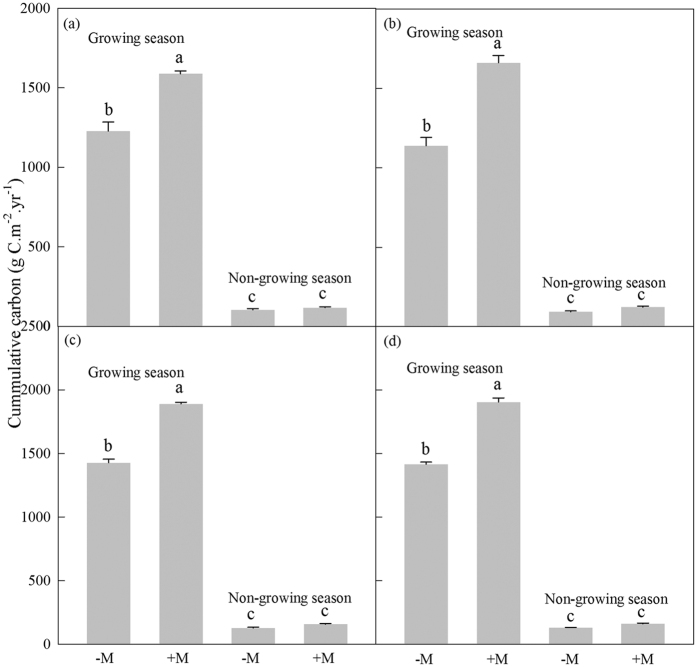
Carbon storage in growing and non-growing season. (**a**–**d**) Indicated wild cherry, cerasus humilis, shiny leaf Yellowh orn, and apricot, respectively. Bar represented means ±  SE. −M and +M denoted without and with AMF inoculation. Different lowercase letters indicated that −M and +M were significantly different at 5% level by LSD.

**Figure 4:**
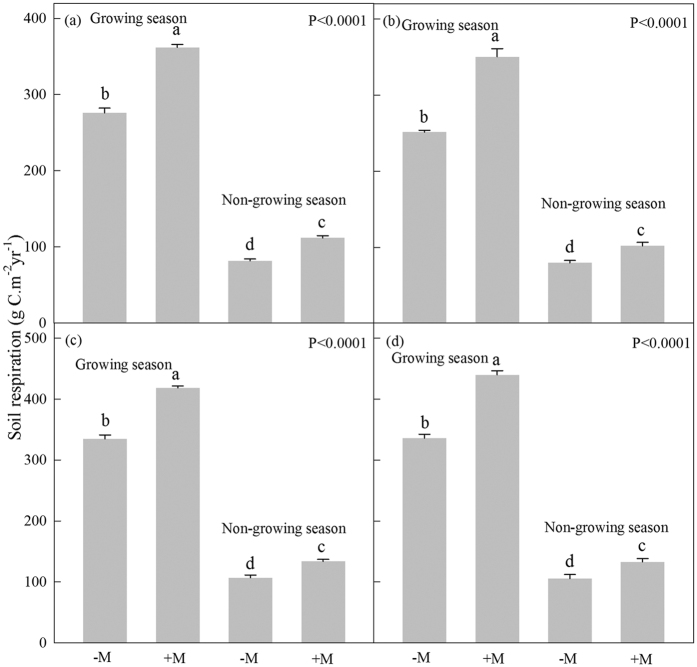
Soil carbon releases via R_s_ in growing and non-growing seasons. (**a**–**d**) Showed that wild cherry, cerasus humilis, shiny leaf Yellowh orn, and apricot, respectively. Bar represented means ± SE. −M and +M denoted without and with AMF inoculation, respectively. Different lowercase letters indicated that −M and +M were significantly different at 5% level by LSD.

Annual cumulative carbon in AMF treated wild cherry, cerasus humilis, shiny leaf Yellowh orn, and apricot were about 1706, 1784, 2049 and 2065 g C.m^−2^.yr^−1^ (Fig. 3a–d). Compared to the corresponding controls, the annual cumulative carbon levels in the four tree species were increased by 28.3%, 44.6%, 31.8% and 33.5%, respectively (Fig. 3a–d). In growing season, AMF increased the carbon storage by 29.4%, 45.7%, 32.4% and 34.4% (*P* < 0.0001) (Fig. 3a–d). AMF also significantly increased the annual cumulative R_s_ carbon in the four tree species by 32.4%, 36.6%, 25.2% and 29.9%, when compared to the corresponding controls (*P* < 0.0001) (Fig. 4a–d).

### Soil temperature sensitivity to R_s_

**Figure 5:**
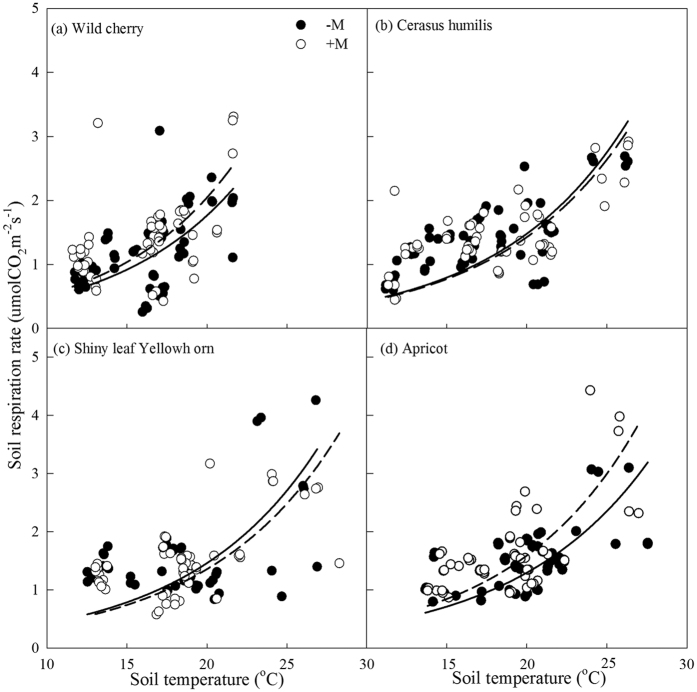
Exponential models of R_s_ based on soil temperature. Soil temperature was plotted against R_s_ for plant species with or without AMF inoculation (n = 36). (**a**–**d**) Indicated that wild cherry, cerasus humilis, shiny leaf Yellowh orn, and apricot, respectively. Solid and hollow circles represented data from without and with AMF inoculation. Solid and dash lines represented the fitted curve of R_s_ to soil temperature without and with AMF inoculation, respectively.

**Figure 6:**
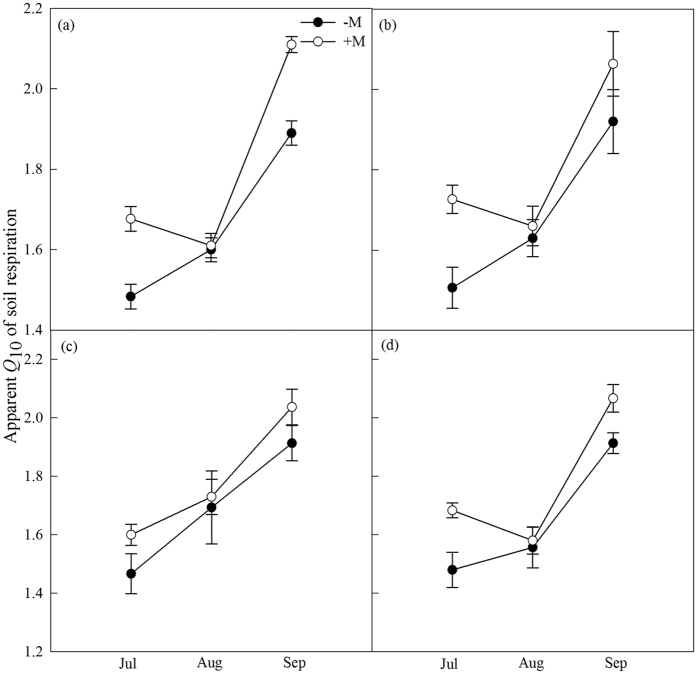
Apparent *Q*_10_ values represents the sensitivity of soil respiration and soil temperature when rises 10 °C. Monthly apparent *Q*_10_ values variation of four tree species without or with AMF inoculation from July to September. Values represented means ± SE. (**a**–**d**) Indicated wild cherry, cerasus humilis, shiny leaf Yellowh orn, and apricot of tree species, respectively.

R_s_ was calculated using exponential function (Eq. 1), temperature dynamic showed over 70% R_s_ variation in AMF treated groups and the correlation of R_s_ showed only 40% variation in control groups (Table 1). Apparent *Q*_10_ and the coefficient *b* were decreased significantly in control apricot and wild cherry (*P* < 0.05) (Table 1; Fig. 5a,d). However, AMF decreased previous two parameters in cerasus humilis and shiny leaf Yellowh orn (Fig. 5b,c). Apparent *Q*_10_ responded differently to temperature (the precipitation in 2014 was higher than 10 year’s average precipitation) in August from that in July or September (Fig. 6a–d).

### Effects of AMF inoculation on carbon-linked parameters

In control of wild cherry, cerasus humilis, shiny leaf yellowh orn, and apricot, the production of OC, EE-GRSP and T-GRSP almost did not change with time (Table 2). In contrast, the production of soil OC, EE-GRSP and T-GRSP in AMF treated of four species increased significantly over time (Table 2). After three months of treatment, the increment of OC, EE-GRSP and T-GRSP in AMF treated groups were all significant (27–37%, 34–45% and 19–22%, respectively (Table 2). After five months of AMF treatments, compared to the control, the levels of OC, EE-GRSP and T-GRSP in AMF treated groups were remarkable enhanced by 52–61%, 55–70% and 36–44%, respectively (Table 2). Collectively, AMF had a positively impacted on soil carbon-linked parameters over time in all of the four tree species (Table 2).

## Discussion

The present study firstly examined photosynthetic ability, R_s_ and carbon storage in respond to AMF inoculation in the coalfields. Under AMF treatment, carbon storage was derived from belowground parts of the tree species. Meanwhile, the temporal variability strongly responded to R_s_ and annual cumulative carbon with exponential models.

### P_n_ and R_s_ responses to AMF inoculation

AMF inoculation incurred a series changes in P_n_, R_s_ and carbon storage via close symbiosis with host plants, leading to enhanced carbon production and improved nutrition status against the detrimental effects^14^,^23^,^24^,^25^,^26^,^27^. Meanwhile, AMF accelerated the organic matter (OM) decomposition rates^28^ and provided some raw materials, which elevated CO_2_ concentration in micro circumstance^29^,^30^ and facilitated soil carbon turnover in the agro-or grassland via CO_2_ stimulation effects^31^. In the moist forest, abundant and diverse AMF (e.g. tropical forests) species created a transient carbon sink via N transferring to reduce carbon lost. This implied that AMF promotes carbon production and storage via increasing P_n_, which ameliorated the negative effect caused by unfavorable environment^32^. Recently, strong evidences showed that the colonized plants have higher P_n_ and ribulose-1,5-bisphosphate carboxylase/oxygenase activity than non-colonized plants, which compensated greater photosynthate^33^,^34^. Carbon was produced directly from P_n_, and AMF perhaps supported the higher metabolism of plants and greater productivity of carbon in the harsh environment^35^,^36^,^37^,^38^,^39^. Furthermore, soil temperature decreasing inhibited substrate movement, lowing microbal activity, which decreased plant P_n_ and belowground carbon allocation. Temperature also reduced P_n_ and the belowground coupling, which potentially affected the substrate availability and carbon cycling^40^. The results demonstrated that AMF can increase P_n_ of the plant, thus increasing carbon allocation.

### Carbon partitioning responses to AMF inocluation

Due to its ability to enhance carbon allocation, AMF has been used in agricultural field and grasslands, but information about carbon storage is omitted in mining soil. Carbon accumulation tended to be higher with AMF inoculation in this study in the growing season. AMF sequestered greater carbon via enhanced P_n_ and photosynthate production, which was transferred from aboveground to roots or microbes. AMF counteracted carbon loss of respiration, due to increase productivity and nutrient acquisition, especially carbon sequestration^35^,^41^. In the non-growing seasons, AMF perferentially allocated photosynthate from tree branchs to belowground parts, increasing the EE-GRSP and T-GRSP of carbon stocks^14^,^38^. Estimately, AMF utilized a large proporation (about 20%) of photosynthates^19^,^42^,^43^,^44^,^45^, leading to more carbon retention via symbiosis^46^,^47^,^48^,^49^,^50^. Consequently, AMF inoculation resulted in remarkable greater carbon sequestration.

### Nonlinear response to R_s_

AMF affected carbon storage and partition, which has been discussed in the previous study^51^. However, no data have shown whether interactions between species and temperature affect AMF facilitated carbon sequestration^52^. As literatures indicated, carbon productivity of the plants was greater with AMF, due to higher availability of nutrients from the soil environments^53^. The beneficial effect of AMF in carbon sequsteration was considered a result of improved availability of nutrients and altered carbon allocation caused by AMF^54^,^55^. The exponential models successfully illustrated the changes of R_s_ in response to soil temperature variation and captured monthly dynamics of all tree species used in the exepriment.

Hysteresis between R_s_ and soil temperature exists in various ecosystems and vegetations^56^,^57^. In general, the decoupling of R_s_ in response to soil temperature is attributed to confound effect, such as precipitation or physiological drought caused hysteresis, which leads to decreased AMF activity and subsequently lower CO_2_ production^57^. Over all, the R_s_-temperature relationship was affected by P_n_, litterfall, and soil microbe activities^58^,^59^,^60^,^61^. However, the detailed mechanisms remain unclear. In this study, due to the existence of hystersis, R_s_ in control groups was higher than AMF inoculation groups under a given temperature, which was in agreement with the previous hypothesis^62^. Most likely, AMF plants accumulated more fresh litter in a given temperature than non-colonized plants. *Q*_10_ was often used to represent the temperature measurement depth in greenhouse or field exprimental models^14^,^63^. In this work, apparent *Q*_10_ increased in AMF treated groups, indicating that AMF increases the sensitivity of R_s_ to soil temperature. Apparent *Q*_10_ value was lower in summer than in cooler season, which might be because of higher substrate availability and severe soil moisture limitation in summer. In consistent with the previous publication^14^, the experiment data showed that R_s_ strongly depends on soil temperature in the study.

Previous studies have used exponential model but ingored the function of AMF^64^. The results suggested that AMF plays a key role in R_s_ and carbon stocks. AMF and soil temperature in combination modulate apparent *Q*_10_ in coalfields.

## Conclusions

This study provided new insights into the impacts of AMF on R_s_, and carbon storage in coalfields. AMF enhances carbon sequestration and the sensitivity of R_s_ to temperature. Higher AMF infection rate significantly enhances R_s_ and P_n_, the positive AMF function is through promoting plant growth, especially increasing leaf area, chlorophyll content, and the *Q*_10_ value. These results clearly demonstrated that AMF infection increased organic matter and glomalin which may be associated with the enhancement of carbon storage in soil. The model of R_s_ to soil temperature should be reassessed to take into account of the interaction between the soil volumetric moisture and mycorrhizal fungus. Thus, further study will focus on long-term AMF effect on carbon stock in a tree species specific manner.

## Materials and Methods

### Study site and experimental set-up

This experiment was conducted at the ecological reclaimed region, which located in Daliuta town (39°18′N, 110°4′E), Shenmu County, Yulin City, Shaanxi Province, Northwest China at 1,200 m height above sea level. This site was a typical junction area of Shanxi, Shaanxi and Inner Mongolia of three Provinces and the south margin of Mu us desert in the loess plateau transition zone. About 70% precipitation falls from June to September, the 10-year (2005–2014) average total precipitation and the potential evaporation were about 150 mm and 2000 mm according to Shenmu Meteorological Station near the experiment site. The experimental location had a typical characteristic of continental climate, with annual average temperature about 8°C. The cumulative temperature above 0 °C and 10 °C were 3,550 °C and 3,210 °C, respectively; the frost-free period is 150 days and total solar radiation is 6,000 MJ m^−2^ year^−1^. The local soil was classified as Aeolian sandy (FAO/UNESCO, 1988) which contained 75% sand, 22% silt, and 3% clay. The topsoil physicochemical properties were: soil OM 4.5 g kg^−1^, total N 0.21 g kg^−1^, Olsen P 5.3 mg kg^−1^, exchangeable K 37.8 mg kg^−1^ and pH value (1:2.5 soil: distilled water) 7.9.

All experiments were carried out in three replicates. The sub-plots of four tree species, wild cherry (*Prunus discadenia Koebne* L.), cerasus humilis *(Prunus dictyneura Diels* L.), shiny leaf Yellow horn (*Xanthoceras sorbifolium Bunge* L.), and apricot (*Armeniaca sibirica* L.) were used in the experiments. Each species was divided into two groups: AMF group and control group. AMF group received the inoculation of AMF at 100 g inoculums/seedling, while the control group did not contain any AMF inoculums.

The four seedlings were obtained from the forest bureau of Shenmu County, Yulin City, Shaanxi Province and Northwest China. Each seedling was transplanted into the plot at temperatures ranging from about 10–15 °C in March 25, 2014 to April 5, 2014. The AMF colonization rate of wild cherry, cerasus humilis, shiny leaf Yellow horn, and apricot were 4.8%, 6.3%, 5.4%, and 7.2% before transplanting and 78.1%, 80.2%, 76.4%, and 81.7% at the end of monitoring stage. Two months after transplantation, over 90% of the seedlings were colonized with mycorrhizal fungus.

Each plot had an area of 20 × 12 m^2^, which consisted of 6 rows, with 10 seedlings per row at 2 m row spacing. 100 g AMF was inoculated in root of AMF plants at seedling transplantation. *Funneliformis mosseae* BGCXJ01 inoculums (Supplied by Beijing Academy of Agriculture and Forestry Sciences) were propagated on *Trifolium repens* (clover) for 12 weeks. 100 g inoculums included spores (10–20 spores/g), colonized root fragments (40 root fragments per gram inoculums, 85% root colonization), and external mycelium^65^.

### R_s_ determination

R_s_ was measured from July to September in individual plot with three replicates. Each measurement event was made in the middle of individual month (about 15^th^) using an Automated Soil Gas Flux System (Li-8100A, Li-cor Inc., Lincoln, Nebraska, USA) coupled with the small PVC chamber (20 cm × 10 cm). The instrument was permanently installed between two rows with identical distance. In each plot, measurements of R_s_ were taken inside three replicate PVC chambers with 20 cm in diameter and 10 cm in height, inserted to a depth of 2–3 cm in soil, capturing respirations. The CO_2_ efflux from this collar mainly derived from soil organic matter decomposition, and root and microbial respiration. Meanwhile, soil temperature and volumetric moisture at the top of 5 cm was measured with the corresponding probes. Specifically, R_s_ was always measured between 8:00 and 12:00 in the morning in the sunny day without wind^66^.

The cumulative R_s_ values were calculated by the method of Bremer *et al.*^67^. Additionally, diel respiration (every 2 hours over 24-hour cycles) was periodically measured in all plots and individual collars. All of the measurements were made in four seasons from 2014 to 2015 due to time constraints. The diel measurements were used to calculate the annual growing season (July to September) cumulative R_s_. Briefly, respiration measured during the daytime was assumed to be the daily maximum soil CO_2_ efflux. Diel measurements were used to calculate the daily minimum efflux as a percentage of maximum efflux. The daily maximum and minimum efflux were used to calculate the average daily efflux. The cumulative flux as the product of average daily flux and the number of days was estimated between each measurement.

### P_n_ determination

Plants assimilated CO_2_ via P_n_. To quantify photosynthetic performance of plant species, the measurement was with two AMF inoculation levels using a portable open system infrared gas analyzer for net CO_2_ assimilation rate of leaves in individual treatment (Li-6400, Li-cor Inc., Lincoln, Nebraska, USA). The experiment was performed from July to September in 2014, including three candidate trees within individual plot. Each measurement event was carried out at 08:00 to 18:00 in sunny day every 2 h, with three fully expanded healthy sun-exposed leaves of each species. Leaves were carefully positioned in the leaf chamber when they were exposed to the 1000 μmol m^−2^ s^−1^ saturating quantum flux level. Parameters including the instantaneous P_n_, transpiration rate (T_r_), stomatal conductance (G_s_), intercellular CO_2_ concentration (C_i_), photosynthetically active radiation (PAR), atmosphere CO_2_ concentration (C_a_), atmosphere temperature (T_a_), leaf temperature (T_leaf_), and relative air humidity (RH) were tested.

Diurnal P_n_ amount was the area which surrounded by the curves of net photosynthetic rate > 0 and the time transverse in the diurnal curves variations of species P_n_. According to the principle, the diurnal net hotosynthetic amount was calculated as follows:


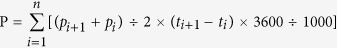


where P was the diurnal total net assimilation amount (mmol.m^−2^.d^−1^), *p*_*i*_ and *p*_*i*+1_ (μmol.m^−2^.s^−1^) was the instantaneous photosynthetic rate of the beginning and next measuring point, respectively; *t*_*i*_ and *t*_*i*+1_ was the time duration of the beginning and next measuring point; n was the numbers of determination times; one hour equaled to 3600 seconds; 1 mmol equaled to 1000 μmol.

Generally, the 20% diurnal assimilation photosynthates production was consumed by dark respiration, and converted the assimilation into the CO_2_ diurnal sequestration as follows:


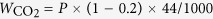


where 44 was CO_2_ mole mass; 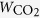 was CO_2_ sequestration mass in the per unit leaf area (g.m^−2^.d^−1^).

According to the P_n_ reaction equation CO_2_ + 4H_2_O → CH_2_O + 3H_2_O + O_2_, which the formula can be calculate the daily CO_2_ absorption in the per unit land area of per plant as follow:


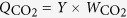



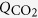
 was the daily CO_2_ sequestration in the per unit land area of per plant species (g.m^−2^.d^−1^).

The average CO_2_ uptake per day of individual plants as follow:


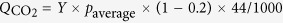



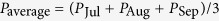


where 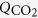 was the daily average CO_2_ sequestration of each plant (g.d^−1^); *P*_average_ was the daily assimilation in the per unit land area (mmol.m^−2^.d^−1^); *Y* was the total leaf area of each plant (m^−2^).

### Soil OC, EE-GRSP and T-GRSP fractions

Soil samples were collected from 0–20 cm of the profile using an auger (35 mm diameter) in each corresponding monitoring stage in 2014. Three cores were collected from individual plot and combined to give one composite sample per plot of each tree species. The composite soil samples were arid-dried and sieved through a 2 mm mesh and placed in plastic bags for chemical analysis.

A simple method for routine determination of soil OC by a modified Mebius procedure was described. It involved a digestion of the soil sample with an acidified dichromate (K_2_Cr_2_O_7_-H_2_SO_4_) solution for 30 minutes in a Pyrex digestion tube in (a) 40 tube block digester preheated to 170 °C and (b) estimation of the un-reacted dichromate by titration of the cooled digest with an acidified solution of ferrous ammonium sulfate using the *N-phenylanthranilic* acid as an indicator.

0.25 g composite sample was extracted with 2 ml of extractant. EE-GRSP was extracted with 20 m*M* citrate solution; pH 7.0 at 121 °C for 30 min. T-GRSP was extracted with 50 m*M* citrate solution, pH 8.0 at 121 °C. 90 min was required for one soil sample and six additional sequential extractions. For the sequential extractions, the supernatant was removed by centrifugation at 10,000 × g for 5 min, 2 ml of 50 mm citrate, pH 8.0 was added to the residue, and samples were autoclaved for 60 min. Extraction of a sample continued until the supernatant showed no red-brown color typical of glomalin. Extracts from each replicate were pooled and then analyzed.

Citrate extractants were added to soil samples and disrupted by a brief (3 min) autoclave cycle. When necessary, the extractant was adjusted with HCl solution until the pH stabilized at 7.0 for 20 m*M* citrate or 8.0 for 50 mM citrate solution. Samples were then subjected to 121 °C for 90 min to extract T-GRSP or 30 min to extract EE-GRSP.

After extraction cycles completed, samples were centrifuged to remove soil particles (10,000 × g for 5 min), and protein in the supernatant was determined by the Bradford dye-binding assay with bovine serum albumin as standard. Concentration of glomalin was extrapolated to mg/g of soil particles by correcting the dry weight of coarse fragments >0.25 mm included in the weight of aggregates and the volume of extractant.

### Data analysis

To assess the four plant species with and without AMF inoculation treatments to long-term exposure and potentially elucidate the role of AMF in previous response, the relationship of R_s_ and P_n_, R_s_ and soil temperature, and carbon storage and AMF inoculation of the key issues have been a focus of this study.

The experiment was at two AMF inoculation levels with four plant species. To determine main effects of AMF inoculation on soil temperature and R_s_ variables, mixed model restricted maximum likelihood estimation with repeated measures was used (PROC MIXED; version 8.0; SAS Institute Inc., Cary, NC, USA, 2003). Turkey’s HSD multiple comparison was used to identify the differences between two AMF inoculation levels.

An exponential model was used to calculate soil temperature sensitivity on R_s_^68^,^69^.


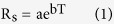


where R_s_ is soil CO_2_ efflux (μmol m^−2^ s^−1^), T is soil temperature (°C) at 0–5 cm depth, a is the basal R_s_, b is temperature sensitivity of CO_2_ efflux. The respiratory quotient (*Q*_10_) was calculated as *Q*_10_ = e^10b^.

Regression coefficients, correlations, figures and curves were obtained by the Sigma-Plot software package (version 11.0, San Jose, California, USA). Analysis was performed to reveal the differences of two AMF inoculation levels with the relevant parameters; differences in means were revealed by LSD (*P* < 0.05) with SAS software package. To determine the effect of AMF on photosynthetic parameters, the data set was divided into two parts; the results were insensitive to variation in species groupings as the sample size was not unduly restricted. Differences in slopes were determined by a dummy variable representing the interaction between independent variable (AMF inoculation level) and plant species in the regression analyses.

### Cumulative carbon calculations

To assess the effect of soil volumetric moisture on carbon accumulation, the cumulative carbon (Cumulative-C) was fitted using a combined exponential and quadratic function as follows:


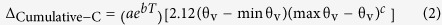


where θ_v_ is the volumetric moisture content (the minimum soil volumetric water content of our data set was 3.1% and the maximum was 37.6%, respectively) and c is the coefficient for soil moisture.

## Additional Information

**How to cite this article**: Wang, Z.-G. *et al.* Arbuscular mycorrhizal fungi enhance soil carbon sequestration in the coalfields, northwest China. *Sci. Rep.* **6**, 34336; doi: 10.1038/srep34336 (2016).

## Figures and Tables

**Table 1:** The exponential model of *Q*_10_ value.

Species	−M			+M		
	*SR*	*r*^*2*^	*Q*_10_	*SR*	*r*^*2*^	*Q*_10_
Wild cherry	1.18*e*^*0.05T*^	0.42	2.13	1.09*e*^*0.09T*^	0.73	2.67
Cerasus humilis	1.21*e*^*0.04T*^	0.49	2.01	1.28*e*^*0.05T*^	0.69	2.51
Shiny leaf Yellowh orn	1.27*e*^*0.05T*^	0.39	1.87	1.31*e*^*0.08T*^	0.75	2.58
Apricot	1.35*e*^*0.07T*^	0.44	1.63	1.42*e*^*0.07T*^	0.71	2.61

Exponential relationship between the R_s_ and soil temperature (T °C; July to September) and apparent *Q*_10_ values.

**Table 2:** Different soil carbon-linked fractions variations.

Indicator	Duration (Month)	Wild cherry		Cerasus humilis		Shiny leaf Yellowh orn		Apricot	
		−M	+M	−M	+M	−M	+M	−M	+M
OC	0	2.36aA	2.35cA	2.42aA	2.39cA	2.53aA	2.48cA	2.44aA	2.46cA
(g kg^−1^)	3	2.42aB	3.18bA	2.51aB	3.27bA	2.65aB	3.35bA	2.51aB	3.13bA
	5	2.55aB	3.67aA	2.63aB	3.84aA	2.67aB	3.76aA	2.56aB	3.83aA
EE-GRSP	0	313aA	319cA	298bA	305cA	313aA	321cA	318aA	325cA
(mg kg^−1^)	3	329aB	428bA	332aB	443bA	321aB	439bA	339aB	453bA
	5	333aB	496aA	339aB	518aA	327aB	508aA	341aB	512aA
T-GRSP	0	897aA	903cA	916aA	904cA	889aA	901cA	898aA	883cA
(mg kg^−1^)	3	915aB	1087bA	923aB	1078bA	908aB	1093bA	923aB	1077bA
	5	936aB	1231aA	945aB	1249aA	926aB	1287aA	930aB	1272aA

Note: values are means of three replicates. Values followed by the small lowercase letters are not significantly different among different inoculation durations (month) of one tree species with or without AMF (+M or −M) at the 5% level by LSD (vertical comparisons); values followed by the same capital letters are not significantly different between −M and +M at the same durations within the same tree species at the 5% level by LSD (horizontal comparisons) in the identical durations (months). −M, +M represented without and with added mycorrhizal fungi inoculums at 100 g per tree species.

Effect of added AMF inoculums on soil OC, EE-GRSP, T-GRSP in four tree species with three inoculation durations.
